# Outdoor recreation self-efficacy among campers: an exemplary application

**DOI:** 10.3389/fpsyg.2026.1825558

**Published:** 2026-05-26

**Authors:** Taner Yılmaz

**Affiliations:** Department of Physical Education and Sport, Faculty of Sports Sciences, Uşak University, Uşak, Türkiye

**Keywords:** camping, outdoor recreation, physical activity, recreational behavior, self-efficacy

## Abstract

**Introduction and objective:**

While the fast-paced and productivity-oriented structure of modern life distances individuals from the natural environment, leisure activities, particularly outdoor recreation, play a vital role in supporting physical and psychosocial well-being. Camping, as a common outdoor recreational activity, involves uncertainty, risk, and personal responsibility, making self-efficacy a key factor in developing safe, conscious, and sustainable behaviors. Despite its importance, practice-based studies focusing on self-efficacy among campers remain limited. Therefore, this study aimed to examine campers’ self-efficacy levels within the context of outdoor recreation and to investigate their relationships with demographic characteristics, physical activity habits, camping experience, and socioeconomic variables.

**Methods:**

This descriptive and correlational study collected data through face-to-face surveys from 317 volunteer campers in Antalya, Muğla, and İzmir. The Outdoor Recreation Self-Efficacy Scale was used for data collection. Independent sample t-tests were conducted according to gender, educational status, and regular exercise habits, while correlation, regression, and one-way ANOVA analyses were performed based on age, income level, and camping experience.

**Results:**

The results showed that male participants had higher self-efficacy levels than female participants. Age was weakly but significantly associated only with the skills/competence sub-dimension. University graduates demonstrated significantly higher self-efficacy than high school graduates. Regular exercise emerged as one of the strongest predictors, showing large effect sizes across all sub-dimensions and total scores. Moreover, self-efficacy increased significantly with greater camping experience. Income level also had a small to moderate effect on self-efficacy.

**Conclusion:**

The findings indicate that self-efficacy in camping and outdoor recreation is multidimensional, experience-dependent, and context-sensitive. These results offer valuable implications for promoting safe and sustainable camping practices, developing inclusive outdoor programs, and enhancing the psychosocial benefits of recreational participation.

## 1 Introduction

The modern lifestyle, with its focus on speed, productivity, and consumption, is gradually distancing individuals from their natural environment and their own inner balance. In this fast-paced lifestyle, individuals seek to make more meaningful and functional use of their leisure time to maintain their physical, psychological, and social well-being. In this regard, recreation is considered a significant area of life that offers opportunities for rest, renewal, and self-actualization through activities chosen voluntarily by the individual outside of their mandatory responsibilities (Torkildsen, 2012; Page and Connell, 2020). Today, the orientation toward outdoor recreation time experiences is becoming an increasingly prominent trend.

Outdoor recreation encompasses activities that individuals engage in within natural environments, utilizing their knowledge, skills, and physical abilities, with outcomes largely dependent on individual preparation, experience, and perceptions (Priest and Gass, 2018). These activities provide multidimensional benefits such as stress reduction, enhanced psychological well-being, improved physical fitness, and a deeper connection with nature (Ewert et al., 2014; Houge Mackenzie and Brymer, 2018). In addition, outdoor recreational activities take individuals out of their familiar environment and expose them to experiences involving uncertainty, risk, and personal responsibility (Ewert and Sibthorp, 2014).

Camping, one of the most common activities in outdoor recreation, offers individuals opportunities to physically and psychologically distance themselves from city life, connect directly with nature, and develop social interaction (Brooker and Joppe, 2014). In this context, camping represents a particularly suitable setting for examining outdoor recreation self-efficacy, as it involves direct interaction with natural environments, requires individual decision-making, and includes elements of uncertainty, risk management, and self-regulation. Camping is a multifaceted activity that can be carried out using tents, caravans, or other forms of accommodation, and has both recreational and tourist aspects. The relatively low cost of accommodation and general vacation expenses makes camping an accessible and economical vacation alternative for different income groups. In recent years, increasing tourism costs and changing holiday preferences after the pandemic have further increased the interest in camping (Margaryan and Fredman, 2017). These features make camping a common form of outdoor recreation for large masses.

However, the nature of camping activities involves physical exertion, environmental conditions, and various risk factors, making individuals’ knowledge, experience, and behavioral characteristics critical. Accidents, environmental damage, and safety issues encountered in campsites are often associated with individuals’ inability to accurately assess risks, insufficient environmental responsibility awareness, and limitations in their perceptions of self-efficacy (Eagleston and Marion, 2017; Stevenson et al., 2019). At this point, the concept of self-efficacy comes to the fore as one of the main psychosocial variables that determine the quality of the camping experience.

Self-efficacy refers to the individual’s belief in their own competence, that they can successfully exhibit the necessary behaviors in a given situation. In the context of outdoor recreation, self-efficacy is directly related to an individual’s capacity to manage challenges they may encounter, accurately perceive risks, and develop safe, environmentally conscious behaviors (Ewert and Sibthorp, 2014). It is stated that individuals with high self-efficacy exhibit more controlled, conscious, and sustainable behaviors in natural environments involving uncertainty, while individuals with low self-efficacy perceptions are more likely to experience negative experiences (Pajares and Schunk, 2001).

Although the concept of self-efficacy has been comprehensively addressed in the literature across many fields, such as education, health, sports, and psychology, there appears to be a limited number of application-based studies in the context of camping and outdoor recreation. Existing studies have mostly examined the effect of self-efficacy on individual experiences within the framework of outdoor education, adventure recreation, and risk perception. Studies addressing self-efficacy levels specifically among individuals who go camping have been relatively limited (Jones and Hinton, 2007; Mittelstaedt and Jones, 2009). However, with the rapid increase in participation in camping today, determining individuals’ levels of self-efficacy is important in terms of promoting safe camping practices, strengthening environmental responsibility, and ensuring the sustainable use of natural areas.

This study aimed to examine the self-efficacy levels of campers in the context of outdoor recreation and to reveal the effects of these levels on camping experiences. The study approaches camping not merely as a leisure activity, but as a form of recreation involving risk, responsibility, and environmental interaction, and evaluates it from the perspective of individual competencies. The distinctive value of this study lies in its examination of self-efficacy in outdoor recreation among campers in the Turkish context and its discussion of the camping experience in terms of individual perceptions, risk management, and sustainability. The findings are expected to contribute to the literature on recreation areas in terms of practical application, the development of conscious and safe camping practices, and the protection of natural areas.

## 2 Materials and methods

### 2.1 Research model

This study employed a descriptive and correlational research design. The aim was to describe outdoor recreation self-efficacy levels among campers and to examine the relationships between self-efficacy and selected demographic and behavioral variables. No experimental manipulation was applied, and the study does not imply causal inference.

### 2.2 Participants and data collection procedure

The sample of this study consists of participants in outdoor recreation activities who engage in camping in the provinces of Antalya, Muğla, and İzmir. Within the scope of the study, face-to-face surveys were conducted with volunteers over the age of 18. Convenience sampling was preferred in the sample selection. These provinces, where camping activities are carried out intensively, enabled access to participants from different age and gender groups.

The recruitment process was conducted through direct contact with campers at selected camping sites in the provinces of Antalya, Muğla, and İzmir. Participants were approached during active recreational periods by trained research assistants, and participation was voluntary. No randomization was applied due to the field-based nature of the study, and convenience sampling was used to reach accessible participants in natural camping environments.

A total of 348 volunteer campers were initially reached. Of these, 340 agreed to participate and completed the questionnaire. After excluding 23 questionnaires due to incomplete or incorrectly filled responses, the final valid dataset consisted of 317 participants.

**Figure 1:**
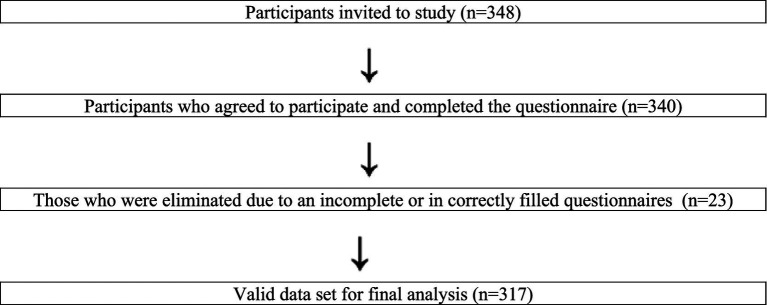
Flow diagram illustrating participant recruitment, eligibility assessment, exclusion process, and final sample included in the study.

Incomplete or inconsistent questionnaires were excluded from the dataset using listwise deletion to ensure data integrity and consistency in statistical analyses (Figure 1).

The study data were collected between May 15 and August 31, 2025, following ethics committee approval from the Uşak University Social Sciences and Humanities Scientific Research and Publication Ethics Committee (dated 13.05.2025, numbered 2025-123). The purpose of the study was explained to the participants, and informed consent was obtained in accordance with the principles of confidentiality and voluntariness.

Data collection was conducted at active camping sites during daytime recreational periods, and participants were approached individually in common recreational areas (e.g., campsite entrances, communal activity zones). No incentive or compensation was provided for participation.

To improve transparency and replicability, the recruitment protocol followed a standardized procedure: trained researchers approached every nth available camper encountered during data collection periods to reduce interviewer selection bias.

### 2.3 Data collection tools

In this study, a specially developed measurement tool was used to assess participants’ levels of self-efficacy in outdoor recreation.

#### 2.3.1 Outdoor recreation self-efficacy scale (ORSE)

The study data were collected through face-to-face interviews using the Outdoor Recreation Self-Efficacy Scale (ORSE), developed by Mittelstaedt and Jones (2009) and adapted into Turkish by Dinç and Tez (2019), with validity and reliability studies conducted. ORSE consists of 2 sub-dimensions and a total of 17 items. These are;

1 Enjoyment/accomplishment: 10 items (items 1–10)
2 Skills/competence: 7 items (items 11–17)

Participants will mark each statement on a Likert-type decimal rating scale ranging from Not at all true (0) to Very true (Stevenson et al., 2019). Although the scale works according to the total score, each sub-dimension can be evaluated independently. High scores indicate a high tendency for self-efficacy.

Validity and reliability analyses of the Turkish form showed that the model achieved perfect and sufficient fit indices (*χ*^2^/sd: 2.38, AGFI: 0.94, CFI: 0.96, GFI: 0.91, RMSEA: 0.07). The factor loadings of the scale were found to be in the range of 0.52–0.89. Cronbach’s Alpha internal consistency coefficients ranged from 0.93 (skills/competence) to 0.83 (enjoyment/accomplishment). Spearman-Brown and Guttman correlation coefficients were 0.79–0.80 for odd numbers; 0.78–0.82 for even numbers.

Participants were classified as “regular exercisers” if they reported engaging in at least 150 min of moderate-intensity physical activity per week, in line with widely accepted public health guidelines.

Camping experience was operationalized based on self-reported frequency of camping participation (number of camping trips in the last 12 months), and categorized into low, moderate, and high experience groups.

Income status was measured using a self-reported categorical scale (low, medium, and high), based on participants’ perceived monthly income level rather than exact numerical values, to increase response accuracy and reduce non-response bias.

### 2.4 Data analysis

All statistical analyses were performed using SPSS version 25.0 (IBM Corp., Armonk, NY, United States). The data analysis process was carried out in three stages:

In the first stage, the dataset was prepared for statistical analysis. This stage includes checking for data entry errors, examining missing data, and calculating the sub-dimension and total scores of the ORSE. No cutoff point was applied on the scale; all analyses were conducted using continuous scores, taking care to preserve statistical power.

Missing data were handled using listwise deletion, as the proportion of missing values was below the acceptable threshold for biased estimation.

In the second stage, the assumptions required for the planned statistical analyses were examined. Distribution normality was evaluated with the Kolmogorov–Smirnov test, and it was observed that all groups met the assumption of normal distribution (*p* > 0.05). Variance homogeneity was checked with the Levene test, and it was observed that variance homogeneity was not achieved in the comparisons of income status and camping experience (*p* < 0.001). Therefore, the Games-Howell *post hoc* test was used for pairwise comparisons. In the comparisons of the two groups (gender, education level, and regular exercise), the independent sample t-test assumptions were checked and provided.

In the third stage, inferential statistical analyses were applied. Independent sample t-tests were used to examine the differences in outdoor recreation self-efficacy levels according to gender, educational level, and regular exercise status, and one-way ANOVA was used to examine the differences according to income status and camping experience. Simple linear regression analysis was performed to examine the predictive power of the age variable on the sub-dimension scores. In all statistical analyses, the significance level (*α*) was set at 0.05. When a significant difference was found with one-way ANOVA, the Games-Howell *post hoc* test was used for pairwise comparisons between groups because variance homogeneity was not ensured.

Effect sizes for independent samples t-tests, one-way ANOVAs, and *post hoc* comparisons were calculated using eta squared (*η*^2^). The *η*^2^ value was calculated using the formula proposed by Kahraman et al. (2024): *t*^2^/(*t*^2^ + N₁ + N₂ − 2). The effect sizes indicate the practical significance of the findings beyond statistical significance and were interpreted according to Cohen (2013) criteria: small (*η*^2^ = 0.01), medium (*η*^2^ = 0.06), and large (*η*^2^ = 0.14).

Although an *a priori* power analysis was not conducted, this limitation has been acknowledged in the manuscript. The final sample size (*n* = 317) is considered adequate for detecting medium effect sizes in comparative analyses. This clarification has been added to improve transparency regarding the statistical adequacy of the study.

## 3 Results

The results are presented in line with the study objectives. First, differences in outdoor recreation self-efficacy across key demographic variables (gender and educational status) are reported. This is followed by analyses of the relationships between self-efficacy and age, exercise behavior, camping experience, and income level using appropriate statistical methods.

Table 1 presents the comparative results of participants’ outdoor recreation self-efficacy levels by gender. The findings show that the mean scores of male participants in the enjoyment/accomplishment sub-dimension were statistically significantly higher than female participants (*t* = −3.320, *p* < 0.001). The effect size of this difference was found to be small (*η*^2^ = 0.034). In the skills/competence sub-dimension, male participants’ self-efficacy scores were found to be significantly higher than those of female participants (*t* = −4.603, *p* < 0.001). The effect size of this difference was found to be moderate (*η*^2^ = 0.063). In terms of the overall ORSE score, it was determined that the outdoor recreation self-efficacy levels of male participants were significantly higher than those of female participants (*t* = −4.047, *p* < 0.001). However, the effect size was small (*η*^2^ = 0.049).

**Table 1:** Outdoor recreation self-efficacy by genders.

Scale/sub-dimension score	Gender	*N*	Mean	SD±	*t*	*p*	*η* ^2^
Enjoyment/accomplishment	Female	146	87.18	8.30	−3.320	<0.001	0.034
	Male	171	89.84	5.36			
Skills/competence	Female	146	61.12	6.28	−4.603	<0.001	0.063
	Male	171	63.81	3.53			
Scale total score	Female	146	148.30	13.99	−4.047	<0.001	0.049
	Male	171	153.65	8.35			

SD, standard deviation; *t* = independent sample *t*-test value; *η*^2^ = effect size (eta squared). Effect size comments: 0.01 = small, 0.06 = moderate, 0.14 = large. *p*-values were reported as <0.001.

Table 2 presents the relationships between age and ORSE sub-dimensions. Accordingly, no significant correlation was observed between the enjoyment/accomplishment sub-dimension and age (*r* = 0.022, *p* = 0.690). In the skills/competence sub-dimension, a small but significant positive correlation was found with age (*r* = 0.121, *p* = 0.032). The correlation between the overall ORSE score and age was not significant (*r* = 0.067, *p* = 0.233). These results show that age has a limited correlation with self-efficacy sub-dimensions. It indicates a small but significant effect, especially in the skills/confidence sub-dimension.

**Table 2:** The relationship between age and outdoor recreation self-efficacy.

Age and sub-dimensions	*r*	*p*
Age and enjoyment/accomplishment	0.022	0.690
Age and skills/competence	0.121*	0.032*
Age and scale total score	0.067	0.233

r = Pearson correlation coefficient; p = significance level (2-tailed). *p < 0.05. *n* = 317.

The results of the simple linear regression analysis presented in Table 3 show that age significantly predicts the skills/competence sub-dimension scores (*B*₁ = 0.084, *t* = 2.16, *p* = 0.032). However, the model’s explanatory power is low (*R*^2^ = 0.015). In other words, the age variable explains only 1.5% of the skills/competence sub-dimension score. This finding suggests that age has a small but statistically significant effect on skills/competence scores.

**Table 3:** Ability to estimate the skills/competence sub-dimension based on age.

Dependent variable	B0	B1	*t*	*p*	*R* ^2^
Skills/competence	60.55	0.084	2.16	0.032*	0.015

B0 = intersection, B1 = regression coefficient (age). *R*^2^ = Explained Variance. *p < 0.05, *N* = 317.

Table 4 presents the comparison results of participants’ outdoor recreation self-efficacy levels according to their educational status. The findings show that the mean scores of university graduate participants in the enjoyment/accomplishment sub-dimension were statistically significantly higher than those of high school graduate participants (*t* = −3.970, *p* < 0.001). The effect size of this difference was found to be small (*η*^2^ = 0.048). In the skills/competence sub-dimension, university graduate participants’ self-efficacy scores were found to be significantly higher than those of high school graduate participants (*t* = −4.745, *p* < 0.001). The effect size of this difference was found to be moderate (*η*^2^ = 0.067). In terms of the overall ORSE score, it was determined that the outdoor recreation self-efficacy levels of university graduate participants were significantly higher than those of high school graduate participants (*t* = −4.782, *p* < 0.001). However, the effect size was moderate (*η*^2^ = 0.068).

**Table 4:** Outdoor recreation self-efficacy according to educational status.

Scale/sub-dimension score	Education level	*N*	Mean	SD±	*t*	*p*	*η* ^2^
Enjoyment/accomplishment	High school	70	85.46	7.82	−3.970	<0.001	0.048
	University	247	89.51	6.47			
Skills/competence	High school	70	60.07	6.08	−4.745	<0.001	0.067
	University	247	63.28	4.64			
Scale total score	High school	70	145.53	13.14	−4.782	<0.001	0.068
	University	247	152.79	10.61			

SD, standard deviation; *t* = independent sample *t*-test value; *η*^2^ = effect size (eta squared). Effect size comments: 0.01 = small, 0.06 = moderate, 0.14 = large. *p*-values were reported as <0.001.

Table 5 presents the comparison results of participants’ outdoor recreation self-efficacy levels according to their regular exercise status. The findings indicate that the enjoyment/accomplishment sub-dimension mean scores of participants who regularly exercised were significantly higher compared to participants who did not exercise (*t* = 7.823, *p* < 0.001). The effect size of this difference was determined to be large (*η*^2^ = 0.163). In the skills/competence sub-dimension, it was determined that the self-efficacy scores of the participants who exercised regularly were significantly higher (*t* = 6.877, *p* < 0.001). The effect size of this difference is large (*η*^2^ = 0.131). In terms of the overall ORSE scores, participants who exercised regularly demonstrated significantly higher outdoor recreation self-efficacy levels compared to those who did not exercise (*t* = 7.833, *p* < 0.001). The effect size of this difference was large (*η*^2^ = 0.163).

**Table 5:** Outdoor recreation self-efficacy according to the status of exercising regularly.

Scale/sub-dimension score	Exercising status	*N*	Mean	SD±	*t*	*p*	*η* ^2^
Enjoyment/accomplishment	Yes	183	91.03	4.77	7.823	<0.001	0.163
	No	134	85.33	8.12			
Skills/competence	Yes	183	64.16	3.52	6.877	<0.001	0.131
	No	134	60.40	6.16			
Scale total score	Yes	183	155.19	7.81	7.833	<0.001	0.163
	No	134	145.72	13.56			

SD, standard deviation; *t* = independent sample *t*-test value; *η*^2^ = effect size (eta squared). Effect size comments: 0.01 = small, 0.06 = moderate, 0.14 = large. *p*-values were reported as <0.001.

As can be seen in Table 6, ORSE scores increase as camping experience increases. In the enjoyment/accomplishment sub-dimension, significant differences were found between the inexperienced group (*X̄* = 80.20, SD = 10.76) and the moderately experienced (*X̄* = 89.25, SD = 4.64) and experienced groups (*X̄* = 91.78, SD = 6.67) [*F*(2, 314) = 47.030, *p* < 0.001, *η*^2^ = 0.230; Games–Howell: 1–2, 1–3]. Differences were also observed between moderately experienced and experienced groups (Games–Howell: 2–3). In the skills/competence sub-dimension, significant differences were found between the inexperienced group (*X̄* = 55.30, SD = 8.12) and the moderately experienced group (*X̄* = 63.13, SD = 3.39) and the experienced group (*X̄* = 65.25, SD = 3.49) in all pairwise comparisons [*F*(2, 314) = 71.800, *p* < 0.001, *η*^2^ = 0.314]; Games–Howell: In terms of the overall ORSE score, significant differences were observed between the inexperienced group (*X̄* = 135.50, SS = 18.33) and the moderately experienced (*X̄* = 152.38, SS = 7.40) and experienced groups (*X̄* = 157.03, SS = 9.34) [*F*(2, 314) = 64.121, *p* < 0.001, *η*^2^ = 0.290; Games–Howell: 1–2, 1–3, 2–3]. These findings suggest that the camping experience has a strong impact on outdoor recreation self-efficacy. Effect sizes indicated a large effect across all sub-dimensions (*η*^2^ = 0.230–0.314).

**Table 6:** Outdoor recreation self-efficacy according to camping experience.

Scale/sub-dimension score	Groups		*n*	*X̄*	SD±	*F*	*p*	Difference (Games–Howell, *p* < 0.05)	Eta squared (*η*^2^)
Enjoyment/accomplishment	1	Inexperienced	40	80.20	10.75	47.030	<0.001	1 < 2 and 1 < 3	0.230
	2	Moderately experienced	213	89.25	4.64			2 < 3	
	3	Experienced	64	91.78	6.67				
	4	Total	317	88.62	6.99				
Skills/competence	1	Inexperienced	40	55.30	8.11	71.800		1 < 2 and 1 < 3	0.314
	2	Moderately experienced	213	63.13	3.39		<0.001	2 < 3	
	3	Experienced	64	65.25	3.49				
	4	Total	317	62.57	5.16				
Scale total score	1	Inexperienced	40	135.50	18.32	64.131	<0.001	1 < 2 and 1 < 3	0.290
	2	Moderately experienced	213	152.38	7.39			2 < 3	
	3	Experienced	64	157.03	9.34				
	4	Total	317	151.19	11.60				

*n* = sample size; *X̄* = mean; SD± = standard deviation; *F* = ANOVA *F*-value; *p* = significance level; *η*^2^ = eta squared (effect size). Groups: 1 = Inexperienced, 2 = Moderately experienced, 3 = Experienced. Difference: Games–Howell *post hoc* test revealed significant differences between the two groups (*p* < 0.05).

As can be seen in Table 7, ORSE scores increase as income level increases. In the enjoyment/accomplishment sub-dimension, significant differences were found between the income less than expenditure group (*X̄* = 86.33, SD = 8.89) and the income equivalent to expenditure group (*X̄* = 88.86, SD = 6.01) and the income more than expenditure groups (*X̄* = 91.65, SD = 4.77) [*F*(2, 314) = 10.590, *p* < 0.001, *η*^2^ = 0.063; Games–Howell: 1–2, 1–3]. In the skills/competence sub-dimension, significant differences were found between the income less than expenditure group (*X̄* = 60.76, SD = 6.54) and the income equivalent to expenditure group (*X̄* = 62.72, SD = 4.52) and the income more than expenditure group (*X̄* = 65.13, SD = 2.84) in all pairwise comparisons [*F*(2, 314) = 13.225, *p* <0.001, *η*^2^ = 0.078; Games–Howell: 1–2, 1–3, 2–3]. A similar pattern was observed in the overall ORSE scores. Significant differences were found between the income less than expenditure group (*X̄* = 147.09, SD = 14.68), income equivalent to expenditure group (*X̄* = 151.58, SD = 10.06) and income more than expenditure group (*X̄* = 156.78, SD = 7.18) [*F*(2, 314) = 12.886, *p* < 0.001, *η*^2^ = 0.076; Games–Howell: 1–2, 1–3, 2–3]. These findings suggest that income status had a *small to medium (borderline)* effect on outdoor recreation self-efficacy (*η*^2^ = 0.063–0.078).

**Table 7:** Outdoor recreation self-efficacy according to income level.

Scale/sub-dimension score	Groups		*n*	*X̄*	SD±	*F*	*p*	Difference (Games–Howell, *p* < 0.05)	Eta squared (*η*^2^)
Enjoyment/accomplishment	1	Income less than expenditure	90	86.33	8.89	10.590	<0.001	1 < 2 and 1 < 3	0.063
	2	Income equivalent to expenditure	173	88.86	6.01				
	3	Income more than expenditure	54	91.65	4.77				
	4	Total	317	88.62	6.99				
Skills/competence	1	Income less than expenditure	90	60.76	6.54	13.225	<0.001	1 < 2 and 1 < 3	0.078
	2	Income equivalent to expenditure	173	62.72	4.52			2 < 3	
	3	Income more than expenditure	54	65.13	2.84				
	4	Total	317	62.57	5.16				
Scale total score	1	Income less than expenditure	90	147.09	14.67	12.886	<0.001	1 < 2 and 1 < 3	0.076
	2	Income equivalent to expenditure	173	151.58	10.05			2 < 3	
	3	Income more than expenditure	54	156.78	7.17				
	4	Total	317	151.19	11.60				

*n* = sample size; *X̄* = mean; SD± = standard deviation; *F* = ANOVA *F*-value; *p* = significance level; *η*^2^ = eta squared (effect size). Groups: 1 = Income is less than expenses, 2 = Income is equal to expenses, 3 = Income is more than expenses. Difference: Pairwise group differences found to be significant by Games–Howell *post hoc* test (*p* < 0.05).

## 4 Discussion

Camping is a multidimensional activity where individuals interact directly with nature, engaging in various experiences on both physical and psychosocial levels. These experiences provide a crucial context for shaping how individuals internalize their self-efficacy perceptions, coping strategies, and societal roles. When self-efficacy is considered as an individual’s belief in their capacity to perform a specific task, it develops dynamically in nature-based activities through interaction with experience, repetition, social support, and individual characteristics.

It is important to note that the empirical base of camping and outdoor recreation self-efficacy research is still relatively fragmented, with most studies focusing on isolated contexts rather than systematically validated measurement frameworks. This limits the accumulation of comparable evidence across recreational settings and highlights the need for more psychometrically robust and context-sensitive studies in future research.

It is important to acknowledge that much of the existing literature on camping and outdoor recreation self-efficacy is based on cross-sectional and correlational survey designs, which inherently limit causal interpretations and do not capture developmental changes in self-efficacy over time (Shea et al., 2019).

In addition to this structural limitation, many studies in the field also lack clear reporting of sample size justification and *a priori* statistical power analysis, which restricts the evaluation of statistical adequacy and the robustness of detected effects.

Furthermore, the widespread use of convenience sampling and voluntary participation procedures increases the risk of self-selection bias, as individuals with higher motivation and prior interest in outdoor activities are more likely to participate. This may lead to systematic overestimation of self-efficacy levels and limits the external validity of findings.

In this regard, the gender variable provides an important perspective in understanding the formation of self-efficacy perception. The current study presents findings consistent with the literature suggesting that male participants’ self-efficacy perceptions were higher than those of female participants (Pomfret and Bramwell, 2016; Melo and Gomes, 2017; Mateer et al., 2021; Krimm, 2022; Rizzolo et al., 2023) his suggests that men’s earlier and more intense exposure to roles socially associated with outdoor space and physical competencies may be a factor that supports their perception of self-efficacy. In contrast, findings in the literature indicate that female participants’ self-efficacy perceptions tend to increase as they gain experience through camping and outdoor activities, benefit from social support mechanisms, and reinforce these perceptions through repeated participation (Whittington and Budbill, 2013; Evans et al., 2020; Smith et al., 2022; Allaire-Duquette et al., 2022).

These results show that gender presents a dynamic structure shaped by interaction with experience, motivation, and social support, rather than a direct and fixed factor on self-efficacy. The present study adds conceptual depth to the literature in terms of evaluating gender differences together with experience and support processes in the context of camping, rather than considering them as individual characteristics.

The age variable stands out as another significant variable in understanding the different dimensions of self-efficacy perception. In this study, no significant correlation was found between age and the enjoyment/accomplishment sub-dimension. On the other hand, a small but significant positive correlation was determined in the skills/competence sub-dimension. This finding suggests that increased experience and practice with age favor the perception of confidence, particularly based on skills. The fact that there is no age-related difference in the enjoyment/accomplishment sub-dimension indicates that this dimension offers a more universal experience area among different age groups.

These findings coincide with studies in the literature that reveal that the correlation between age and self-efficacy varies according to context and sub-dimensions (Aslan, 2017; Demir and Kabakçı, 2023). It is seen that age plays an indirect role, especially in skill-based dimensions that require experience and practice, whereas emotional and motivational dimensions are less sensitive to age. In this regard, the current study revealed that the age variable is not a factor that explains self-efficacy alone, but a process that gains significance with experience.

Educational status emerges as another noteworthy variable in shaping the perception of self-efficacy in the context of camping and outdoor recreation. The fact that university graduate participants had higher scores in both the skills/competence sub-dimension and the overall ORSE scores indicates that education supports the perception of self-efficacy through processes such as problem solving, cognitive flexibility, and openness to learning (Nikbay Arslantaş and Bavlı, 2022). In addition, the observation of a significant difference in the enjoyment/accomplishment sub-dimension based on educational level suggests that the cognitive and psychosocial dimensions of the camping experience also interact with education (Fields, 2010; Hursen and Islek, 2017).

However, the relatively limited impact of this sub-dimension suggests that the enjoyment derived from nature-based activities offers a largely shared and universal experience space (Nikbay Arslantaş and Bavlı, 2022). While the level of education provides a framework that supports the process of making sense of experience and transforming it into skills, it plays a decisive role in the skill-based dimensions of self-efficacy. In this regard, the study contributes to the literature by revealing that educational status shows a small effect in the enjoyment/accomplishment dimension (*η*^2^ < 0.06) and a medium (borderline) effect in the skills/competence and total score (*η*^2^ = 0.06–0.07).

Regular exercise status has been identified as one of the main variables that strengthen the perception of self-efficacy in the context of camping and outdoor recreation. The fact that physically active individuals had higher scores, especially in the skills/competence sub-dimension, suggests that physical readiness has a direct effect on the belief in accomplishing tasks. It can be stated that regular exercise also supports individuals’ psychosocial skills, such as resilience, coping with stress, and risk management, and thus strengthens the perception of self-efficacy (Mielenz et al., 2013; Craike et al., 2018; Myers et al., 2020; Lee et al., 2024).

The higher enjoyment/accomplishment sub-dimension scores of individuals who exercise suggest that physical activity increases satisfaction from nature-based experiences by supporting motivation and emotional regulation processes. These differences reflect a large effect size (*η*^2^ ≥ 0.14), indicating a strong association between regular exercise and self-efficacy. Current findings are consistent with studies showing that physically active individuals have a higher sense of self-efficacy in nature-based activities (Eatough et al., 2015; Mutz and Müller, 2016; Beames et al., 2018; Allan and McKenna, 2019; Phillips et al., 2023; Tyne et al., 2024).

Income status also stands out as an effective variable on access to and gains from camping experiences. In the study, it was observed that participants with high income levels had a higher perception of self-efficacy in the context of camping and outdoor recreation. This situation suggests that economic resources can increase the depth of the experience through elements such as equipment, transportation, and continuity of participation. In particular, the fact that skill-based confidence perception is related to income level supports the role of access to experience and repetition in self-efficacy development.

The literature emphasizes that structured camping and nature-based programs for low-income individuals offer important intervention areas that support self-efficacy development (Yıldız et al., 2016; Richmond and Sibthorp, 2019).

From a methodological standpoint, convenience-based participation in camping programs and voluntary engagement of individuals introduce self-selection bias, meaning that individuals with higher motivation and prior interest are more likely to participate. This can systematically inflate observed self-efficacy levels and limit the generalizability of findings in recreational research contexts (Schremp and Rapport, 2021).

Moreover, the absence of reported sample size calculation and statistical power analysis further limits the methodological rigor of the study, as it prevents a clear assessment of whether the sample size was adequate to detect meaningful effects.

Furthermore, the dominance of cross-sectional designs in the field restricts the ability to understand how self-efficacy evolves as a consequence of repeated exposure to camping experiences. Longitudinal and experimental designs would therefore provide stronger evidence for causal pathways between outdoor recreation participation and self-efficacy development.

In this regard, the current findings reveal that income status shows a small-to-medium effect on outdoor recreation self-efficacy (*η*^2^ = 0.063–0.078) and that these inequalities can be reduced through appropriately designed interventions.

Similarly, the cross-sectional nature of the study limits the ability to determine causality and does not allow for the observation of how self-efficacy evolves through repeated camping experiences over time. This is a well-recognized limitation in psychological and recreational research designs using correlational survey methods (Shea et al., 2019).

## 5 Conclusion

This study demonstrates that outdoor recreation self-efficacy among campers is primarily associated with experiential and behavioral factors, particularly camping experience and regular physical activity. These variables emerged as the strongest associations with self-efficacy, especially in the skills/competence dimension, highlighting the importance of repeated exposure and physical readiness in nature-based environments. Demographic factors such as gender, education, age, and income showed statistically significant but comparatively weaker associations with self-efficacy outcomes.

Overall, camping represents not only a recreational activity but also an experiential learning environment that is linked to individuals’ perceived competence and psychological readiness in outdoor settings. The focus on camping provides a context-specific understanding of self-efficacy in real outdoor environments characterized by experiential learning and situational challenges. These findings underline the importance of promoting regular participation in physical activity and structured camping experiences as factors associated with higher self-efficacy levels in nature-based contexts. However, the use of convenience sampling limits the generalizability of the findings. Future research should employ probability sampling and longitudinal designs to enhance external validity and to examine developmental changes over time.

## Data Availability

The original contributions presented in the study are included in the article/supplementary material, further inquiries can be directed to the corresponding author.
